# Structure of *Bacillus subtilis* γ-glutamyl­transpeptidase in complex with acivicin: diversity of the binding mode of a classical and electrophilic active-site-directed glutamate analogue

**DOI:** 10.1107/S1399004713031222

**Published:** 2014-01-31

**Authors:** Tomoyo Ida, Hideyuki Suzuki, Keiichi Fukuyama, Jun Hiratake, Kei Wada

**Affiliations:** a Department of Biological Sciences, Graduate School of Science, Osaka University, Toyonaka, Osaka 560-0043, Japan; b Division of Applied Biology, Graduate School of Science and Technology, Kyoto Institute of Technology, Goshokaido-cho, Matsugasaki, Sakyo-ku, Kyoto 606-8585, Japan; c Institute for Chemical Research, Kyoto University, Uji, Kyoto 611-0011, Japan; d Organization for Promotion of Tenure Track, University of Miyazaki, Miyazaki 889-1692, Japan

**Keywords:** γ-glutamyltranspeptidase, inhibitors, acivicin, glutamine antagonist, glutathione, glutamine amidotransferase, Ntn-hydrolase family

## Abstract

γ-Glutamyltranspeptidase (GGT) is an enzyme that plays a central role in glutathione metabolism, and acivicin is a classical inhibitor of GGT. Here, the structure of acivicin bound to *Bacillus subtilis* GGT determined by X-ray crystallography to 1.8 Å resolution is presented, in which it binds to the active site in a similar manner to that in *Helicobacter pylori* GGT, but in a different binding mode to that in *Escherichia coli* GGT. In *B. subtilis* GGT, acivicin is bound covalently through its C3 atom with *sp* ^2^ hybridization to Thr403 O^γ^, the catalytic nucleophile of the enzyme. The results show that acivicin-binding sites are common, but the binding manners and orientations of its five-membered dihydroisoxazole ring are diverse in the binding pockets of GGTs.

## Abstract 2

The binding modes of acivicin, a classical and an electrophilic active-site-directed glutamate analogue, to bacterial γ-glutamyltranspeptidases were found to be diverse.

## 1. Introduction   

γ-Glutamyltranspeptidase (GGT; EC 2.3.2.2) is involved in the degradation of γ-glutamyl compounds such as glutathione (GSH; γ-glutamyl-cysteinyl-glycine; Tate & Meister, 1981 ▶; Stole *et al.*, 1990 ▶). A major physiological role of this enzyme is to cleave the extracellular GSH as a source of cysteine for intracellular glutathione biosynthesis (Hanigan & Ricketts, 1993 ▶). Another crucial role of GGT is to cleave glutathione-*S*-conjugates as a key step in the detoxification of xenobiotics and drug metabolism (Taniguchi & Ikeda, 1998 ▶). GGTs are found in all kingdoms of life, but their *in vivo* localization, and perhaps accordingly their physiological roles, differ significantly depending on the organisms; for example, in bacteria GGT is expressed in the periplasmic space or is secreted into the extracellular environment (Tate & Meister, 1981 ▶), while in mammalian cells it is bound to the external surface of the plasma membrane (Taniguchi & Ikeda, 1998 ▶) and in plants it is localized to the apoplast and the vacuole (Ohkama-Ohtsu *et al.*, 2009 ▶). Mammalian GGT orthologues share high sequence identities (*i.e.* human, pig and rat GGTs share ∼80% sequence identity), but in contrast bacterial GGTs share limited sequence homology (∼30%) to one another and have insertion/deletion regions in their sequence. However, the residues involved in the catalysis, substrate/inhibitor recognition and binding of the γ-glutamyl moiety are highly conserved among bacterial GGT orthologues (Okada *et al.*, 2006 ▶, 2007 ▶; Boanca *et al.*, 2007 ▶; Wada *et al.*, 2008 ▶, 2010 ▶; Williams *et al.*, 2009 ▶). Hence, the catalytic/inhibitor-binding reactions by bacterial GGTs are believed to proceed with a common mechanism.

GGT is a heterodimeric enzyme consisting of large (L; ∼40 kDa) and small (S; ∼20 kDa) subunits that are formed from an inactive precursor protein (∼60 kDa) by post-translational and autocatalytic cleavage (Suzuki & Kumagai, 2002 ▶; Okada *et al.*, 2006 ▶, 2007 ▶). The autocatalytic processing requires a specific nucleophilic residue (Thr) that catalyzes the cleavage of the peptide bond just preceding this residue to liberate the catalytic S subunit with this residue at its N-­terminus. In the mature enzyme, this residue becomes the catalytic nucleophile of the enzyme (Suzuki & Kumagai, 2002 ▶) and is critical for the binding of irreversible inhibitors such as acivicin (see below) and phosphonate-based transition-state analogues (Inoue *et al.*, 2000 ▶; Castonguay *et al.*, 2007 ▶; Han *et al.*, 2007 ▶). This maturation process is common to all of the GGTs identified so far and is a characteristic trait of the N-terminal nucleophile hydrolase (Ntn-hydrolase) family, which includes GGT, 20S proteasome and an array of glutamine-dependent biosynthetic enzymes (Gln amidotransferases, GATs; see below) (Brannigan *et al.*, 1995 ▶; Oinonen & Rouvinen, 2000 ▶).

**Figure 1:**
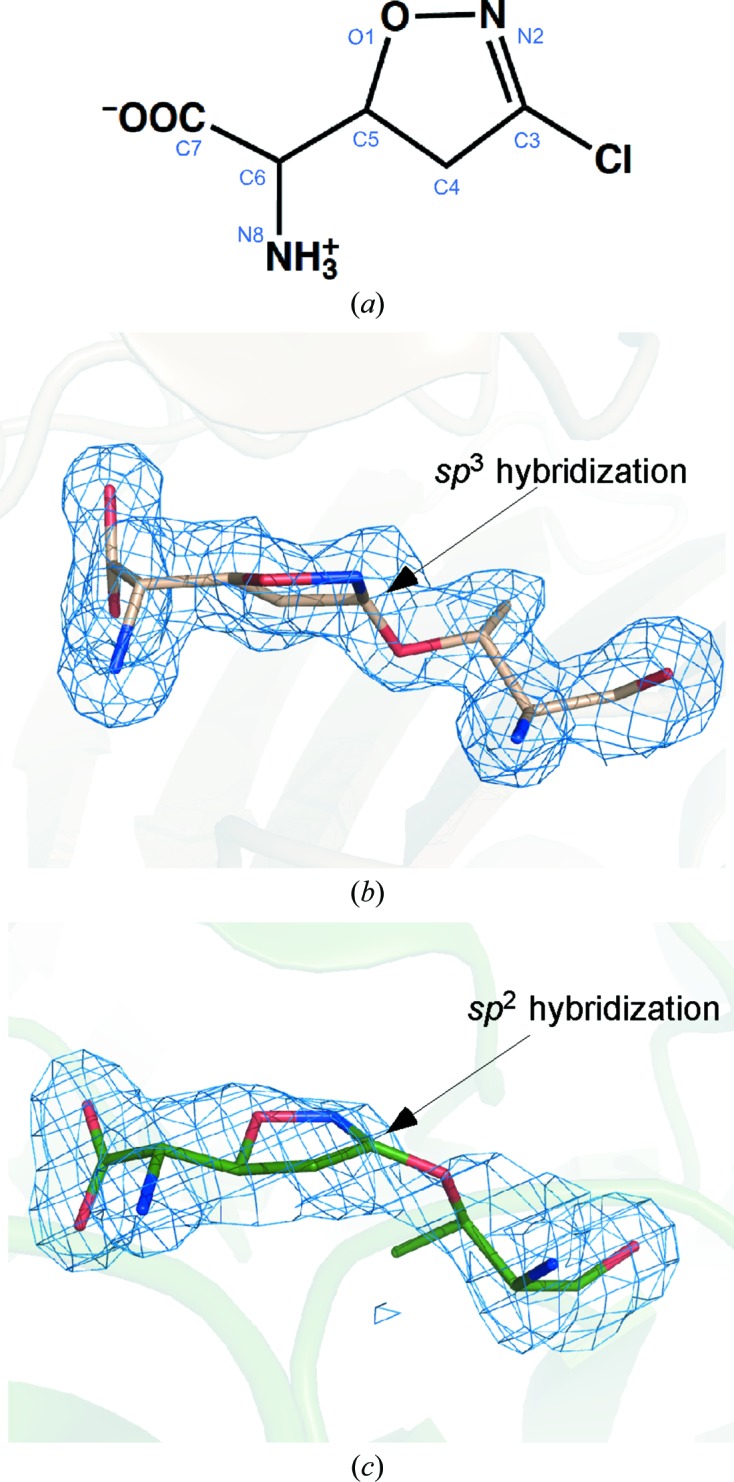
The structure and binding modes of acivicin. (*a*) The structure of acivicin. Previously reported acivicin-binding configurations in (*b*) *E. coli* GGT at 1.65 Å resolution (Wada *et al.*, 2008 ▶) and (*c*) *H. pylori* GGT at 1.70 Å (Williams *et al.*, 2009 ▶). An OMIT *F*_o_ − *F*_c_ map for the acivicin adduct contoured at 2.0σ (blue) is overlaid on its stick model of each GGT and bound acivicin.

Acivicin, a well known glutamine antagonist that was found originally to inhibit GATs (Tso *et al.*, 1980 ▶), also inhibits a wide range of GGTs from mammalian to bacterial enzymes (Fig. 1 ▶ *a*; Stole *et al.*, 1994 ▶; Flahou *et al.*, 2011 ▶) as an electrophilic glutamate analogue. We previously reported the first X-­ray crystal structure of GGT in complex with acivicin (Wada *et al.*, 2008 ▶). In *Escherichia coli* GGT, acivicin is covalently bound to the O^γ^ atom of the catalytic Thr residue (Thr391) through the C3 atom (imino C atom), but contrary to our initial expectations from its chemical structure (an imidoyl chloride) and the anticipated common chemistry (nucleophilic substitution) of acivicin, the C3 atom adopted an *sp* ^3^ hybridization (Fig. 1 ▶ *b*; Wada *et al.*, 2008 ▶). The formation of this structure was explained as a result of migration of the single and double bonds involving opening and re-closure of the dihydroisoxazole ring. However, in a subsequent study with *Helicobacter pylori* GGT, acivicin was reported to bind to the catalytic Thr380 through the C3 atom but with a planar and perhaps *sp* ^2^ hybridization (Fig. 1 ▶ *c*; Williams *et al.*, 2009 ▶). This is apparently the result of a simple and conventional nucleophilic substitution of Cl at the imidoyl C atom by Thr380 O^γ^. The question may arise here as to what has led to the discrepancy in the mode of action of acivicin towards the mutually homologous bacterial GGTs. What chemistry, if any, may lie hidden in the apparently unusual structure of acivicin (an imidoyl chloride in a dihydroisoxazole scaffold with an overall structure analogous to glutamate) and its reaction with GGT?

In our effort to address these questions, we carried out the X-ray crystallographic analysis of a bacterial GGT from *Bacillus subtilis* in complex with acivicin. *B. subtilis* GGT is a 552 amino-acid protein (L, 36–402; S, 403–587; Minami *et al.*, 2003*a* ▶,*b* ▶). Bacterial GGTs share the same overall fold, but *B. subtilis* GGT is unique in that it lacks a lid-loop that covers the bound substrate and has a tail at the C-terminal end of the L subunit (Boanca *et al.*, 2007 ▶; Okada *et al.*, 2007 ▶; Wada *et al.*, 2010 ▶). The conformation of the C-terminal end of the L subunit of *B. subtilis* GGT does not change drastically upon cleavage of the precursor protein (Wada *et al.*, 2010 ▶), unlike *E. coli* GGT (Okada *et al.*, 2007 ▶) or *H. pylori* GGT (Boanca *et al.*, 2007 ▶). Here, we report the binding mode of acivicin to *B. subtilis* GGT at 1.8 Å resolution, showing that acivicin is bound to the O^γ^ atom of Thr403, the catalytic nucleophile of the enzyme, through its C3 atom. The observed electron density around the C3 atom was best fitted to the planar and *sp* ^2^-hybridized C atom, consistent with a simple nucleophilic substitution of Cl at the imino C atom by the O^γ^ atom of Thr403. Furthermore, comparison of three bacterial enzymes, the GGTs from *E. coli*, *H. pylori* and *B. subtilis*, in complex with acivicin showed significant diversity in the orientation of the dihydroisoxazole ring among the three GGTs. The differences are discussed in terms of the recognition of the α-­amino and α-carboxy groups in preference to the dihydro­isoxazole ring, as observed in time-lapse soaking crystal structures of *B. subtilis* GGT with acivicin.

## 2. Materials and methods   

### 2.1. Expression and purification of *B. subtilis* GGT   

The expression and purification of *B. subtilis* GGT have been described previously (Wada *et al.*, 2010 ▶). Briefly, *E. coli* C41(DE3) strain transformed with the plasmid pCold I-His_6_-*ggt* was grown at 310 K in 3.6 l liquid Terrific broth containing ampicillin (50 µg ml^−1^) to an optical density of 0.6 at 600 nm. At this stage, expression of the N-­terminal His_6_-tagged GGT was induced by decreasing the temperature from 310 to 288 K, followed by adding isopropyl β-d-1-thiogalactopyranoside (IPTG) to a final concentration of 1 m*M*. After induction, the transformant was cultured at 288 K for 38 h, the cells were collected by centrifugation (2560*g*) and disrupted. The soluble fraction was subjected to COSMOGEL His-Accept resin (Nacalai Tesque) and the N-­terminal His-tagged GGT was eluted according to the manufacturer’s protocol. Fractions containing the GGT were collected and concentrated. The His_6_-GGT was further purified by gel filtration using a HiPrep 16/60 Sephacryl S-200 HR column (GE Healthcare) to homogeneity as checked by SDS gels stained with Coomassie Blue.

### 2.2. Preparation of the acivicin-bound GGT crystals   

The purified His_6_-GGT solution was desalted by repeated concentration using a Vivaspin filter (GE Healthcare) followed by dilution with 50 m*M* HEPES buffer pH 7.0. We re-screened the crystallization conditions of *B. subtilis* GGT to obtain a new crystal form; the previously obtained crystals had a large unit cell (Wada *et al.*, 2010 ▶). The initial crystallization trials were performed using commercially available sparse-matrix screening kits such as Crystal Screen, Crystal Screen 2, Crystal Screen Lite, Crystal Screen Cryo, Natrix, PEG/Ion, PEG/Ion 2 (Hampton Research), Wizard I–III (Emerald BioStructures) and JBScreen 1–6 (Jena Bioscience). The conditions that produced crystals were optimized by varying the concentrations of protein, the precipitants, the buffer system and the pH. All crystallization trials were carried out using the micro-oil batch method at 293 K. Diffraction-quality crystals were produced when the drop was prepared by mixing 0.9 µl protein solution (10 mg ml^−1^) with 0.9 µl reservoir solution [26%(*w*/*v*) PEG 3350, 0.7 *M* sodium thiocyanate, 6%(*v*/*v*) ethylene glycol] layered under 10 µl Al’s oil (Hampton Research). The crystals grew in a week to typical dimensions of 0.1 × 0.1 × 0.3 mm. Acivicin-bound GGT crystals were obtained by soaking the crystals for 2 h in crystallization solution containing 5 m*M* acivicin.

### 2.3. X-ray data collection of the acivicin-bound GGT crystals   

The acivicin-bound GGT crystals were soaked in a cryoprotectant solution which was prepared by adding 30%(*v*/*v*) ethylene glycol to the reservoir solution and flash-cooled in a nitrogen-gas stream at 100 K. Diffraction data were collected using synchrotron radiation and a Quantum 315 detector (Area Detector Systems) on beamline BL38B1 at SPring-8, Harima, Japan. Each diffraction image was taken by oscillating 0.5° and a total of 400 images were processed; the integrated intensities were merged and scaled using the *HKL*-2000 suite (Otwinowski & Minor, 1997 ▶). The results of the data collection are summarized in Table 1 ▶.

### 2.4. Structure determination and refinement of the acivicin-bound GGT   

Because the acivicin-bound GGT crystals used in this study had entirely different unit-cell parameters compared with the previously obtained crystals (Wada *et al.*, 2010 ▶), we applied the molecular-replacement method to solve the initial phase using the *B. subtilis* GGT structure (PDB entry 3a75; Wada *et al.*, 2010 ▶) as the search probe, where the bound glutamate, water molecules and Thr403 residue were omitted to reduce the model bias in the electron density of the acivicin adduct. Rotational and translational searches of the diffraction data (15.0–4.0 Å resolution) using *MOLREP* (Vagin & Teplyakov, 2000 ▶) from the *CCP*4 package (Winn *et al.*, 2011 ▶) located one molecule in an asymmetric unit. The structure was subjected to rigid-body refinement using data in the resolution range 25–3.0 Å using *REFMAC*5 (Murshudov *et al.*, 2011 ▶). The structure was further refined at 1.8 Å resolution by restrained refinement in *REFMAC*5, and manual model revision was carried out with *Coot* (Emsley & Cowtan, 2004 ▶). The ordered water molecules were added to the model using *ARP*/*wARP* (Perrakis *et al.*, 2001 ▶; Cohen *et al.*, 2008 ▶). The electron-density map at the final stage was clear enough to assign the exact orientation of the bound acivicin. The acivicin molecule was unambiguously fitted to the *F*_o_ − *F*_c_ map of the substrate-binding pocket. Finally, by using the coordinates, topologies and parameters of the acivicin–Thr403 adduct generated by the *PRODRG*2 server (Schüttelkopf & van Aalten, 2004 ▶), positional refinement of the fitted acivicin–Thr403 adduct was performed by 20 cycles of restrained refinement in *REFMAC*5 [the weighting X-ray *versus* geometry (WEIG keyword) was set to ‘auto’ and a standard restraint for the peptide bond between acivicin–Thr403 and Thr404 was defined in the *REFMAC*5 geometry libraries]. Structure-refinement statistics are summarized in Table 1 ▶. The geometry of the final models was analyzed using *PROCHECK* (Laskowski *et al.*, 1993 ▶).

### 2.5. Data deposition   

The coordinates and structure factors have been deposited in the Protein Data Bank as entries 3whq (crystal without soaking), 3whr (GGT crystal soaked in acivicin solution for 3 min) and 3whs (GGT crystal soaked in acivicin solution for 120 min; the acivicin-bound form).

## 3. Results   

### 3.1. Overall structure of the acivicin-bound *B. subtilis* GGT   

The structure of *B. subtilis* GGT in complex with acivicin was refined at 1.8 Å resolution to *R* and *R*_free_ values of 0.183 and 0.209, respectively. The asymmetric unit contains one heterodimeric GGT molecule, which binds one acivicin at the catalytic site. Although the electron density for GGT was mostly of high quality and continuous, the densities for the N-terminal His-tag segment and residues 396–402, corresponding to the C-terminus of the L subunit, were poorly defined; accordingly, these residues were not included in the model. The topology of the acivicin-bound GGT has a stacked αββα core structure comprising two central β-sheets and surrounding α-­helices, which is identical to those of the substrate-free form of *E. coli* GGT (PDB entry 2e0x; Okada *et al.*, 2007 ▶) and the glutamate-bound *E. coli* GGT (PDB entry 2dbx; Okada *et al.*, 2006 ▶).

### 3.2. Binding mode of acivicin in *B. subtilis* GGT   

**Figure 2:**
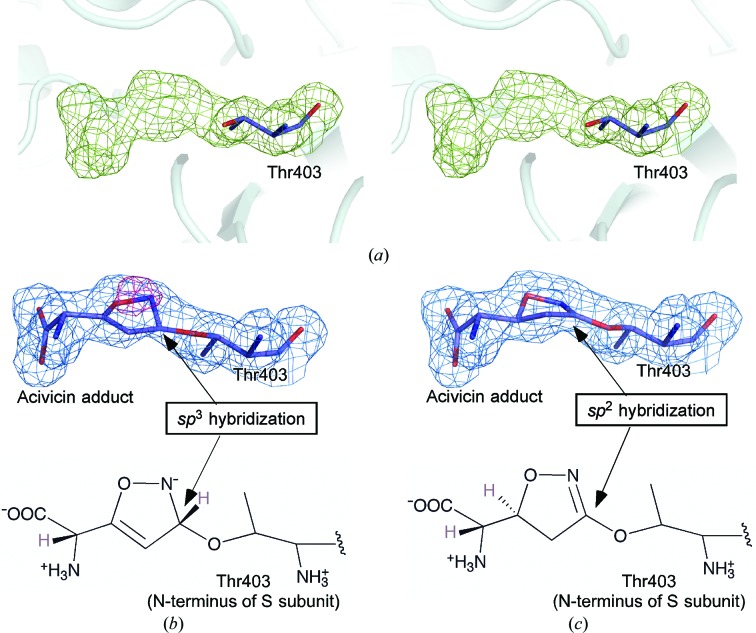
Validation of the binding mode of acivicin toward *B. subtilis* GGT. (*a*) Stereoview of the unbiased *F*_o_ − *F*_c_ map for the acivicin adduct contoured at 2.0σ (green). This map was obtained at the final stage of the refinement for the GGT moiety lacking Thr403 and the acivicin molecule. (*b*) The bound acivicin model assuming that acivicin is bound to the C3 atom with *sp* ^3^ hybridization. (*c*) The *sp* ^2^-hybridized model. The 2*F*_o_ − *F*_c_ map at 1.0σ (blue) and *F*_o_ − *F*_c_ map at 3.0σ (red) are overlaid on the stick models of the acivicin adduct in the GGT structure.

The electron-density map of acivicin-bound GGT revealed that acivicin was bound to the substrate-binding pocket. As expected, the imino C atom (C3 atom) of the dihydroisoxazole ring of acivicin was linked by a covalent bond to the O^γ^ atom of Thr403, the catalytic nucleophile of *B. subtilis* GGT (Fig. 2 ▶ *a*). Two previous structures of bacterial enzymes in complex with acivicin showed distinct differences in the hybridization of the C3 atom; in *E. coli* GGT acivicin was bound to the enzyme through the C3 atom in a tetrahedral configuration with an *sp* ^3^ hybridization, whereas in *H. pylori* GGT acivicin was bound to the enzyme through the same C atom (C3 atom) but in a different configuration, possibly with an *sp* ^2^ hybridization. To define the hybridization of the C3 atom of acivicin bound to *B. subtilis* GGT, we made two models, an *sp* ^3^-hybridized model and an *sp* ^2^-hybridized model, and their residual electron densities around acivicin were assessed (Figs. 2 ▶ *b* and 2 ▶ *c*). When the *sp* ^3^-hybridized model, in which the former imino C atom and the surrounding atoms of acivicin adopt a tetrahedral configuration, was fitted to the electron-density map, a significant residual electron density was observed (Fig. 2 ▶ *b*). In contrast, the *sp* ^2^-hybridized model nicely fitted to the density (Fig. 2 ▶ *c*). Hence, we concluded that acivicin was bound to the O^γ^ atom of Thr403 of *B. subtilis* GGT with the C3 atom adopting an *sp* ^2^ hybridization (O1—N2=C3—O^γ^ torsion angle, 175.9°; C4—C3—O^γ^—C^β^ torsion angle, 179.0°; C4—C3—O^γ^ bond angle, 123.0°; N2=C3—O^γ^ bond angle, 126.6°; C4—C3 =N2 bond angle, 110.2°). Similarly, examination of the hybridization at the C5 atom of the dihydroisoxazole ring confirmed that the C5 atom adopted a tetrahedral configuration as anticipated from its original *sp* ^3^ hybridization (C7—C6—C5—O1 torsion angle, 76.8°; N8—C6—C5—C4 torsion angle, 50.9°; C6—C5—O1 bond angle, 112.4°; C6—C5—C4 bond angle, 114.3°; C4—C5—O1 bond angle, 102.0°). Consequently, the reaction of acivicin with the active-site O^γ^ atom of Thr403 of *B. subtilis* GGT is best explained by simple nucleophilic substitution of Cl at the imino C atom (C3 atom) without a concomitant change in the hybridization as observed for the *E. coli* GGT complex. A longer soaking time gave essentially the same result (data not shown).

### 3.3. Comparison of the binding mode of acivicin among GGTs   

**Figure 3:**
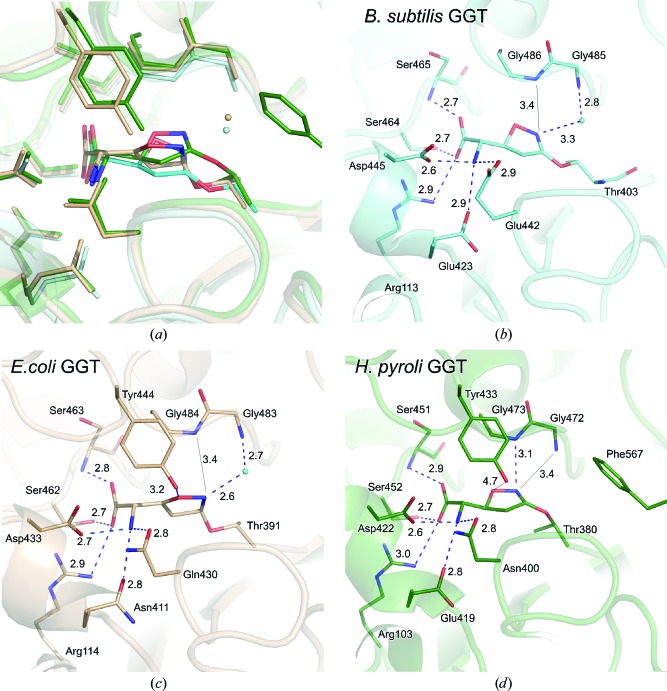
The surrounding environments of the acivicin adduct. (*a*) Superimposition of the acivicin-bound GGTs of *B. subtilis* (cyan), *E. coli* GGT (light brown) and *H. pylori* GGT (green). The acivicin adduct-surrounding residues of (*b*) *B. subtilis* GGT, (*c*) *E. coli* GGT (Wada *et al.*, 2008 ▶) and (*d*) *H. pylori* GGT (Williams *et al.*, 2009 ▶). The blue broken lines indicate hydrogen bonds and the black lines indicate the distances between two atoms without hydrogen bonds. The distances are represented in Å.

Owing to the *sp* ^2^ hybridization, the covalent bond angle between Thr403 O^γ^ of *B. subtilis* GGT and the acivicin C4 atom is more similar to that in *H. pylori* GGT (C3 in *sp* ^2^ hybridization) than to that in the *E. coli* GGT complex (C3 in *sp* ^3^ hybridization). However, careful examination of the complex structures identified significant differences in the orientations of the dihydroisoxazole ring among the three GGTs (Fig. 3 ▶ *a*). When the catalytic pockets of *B. subtilis*, *E. coli* and *H. pylori* GGTs were compared, the environments around the α-­amino and α-carboxy moieties of acivicin were similar to each other; the key amino-acid residues that hydrogen bond to the α-­amino and α-carboxy groups were conserved (Figs. 3 ▶ *b*, 3 ▶ *c* and 3 ▶ *d*). These hydrogen-bond networks including salt bridges were also observed for the α-­amino/α-carboxy groups of the γ-glutamyl-enzyme intermediate in the *E. coli* GGT complex (Okada *et al.*, 2006 ▶) and the glutamate (the enzymatic reaction product) in both the *H. pylori* GGT (Boanca *et al.*, 2007 ▶) and the *E. coli* GGT complexes (Boanca *et al.*, 2007 ▶; Okada *et al.*, 2007 ▶; Wada *et al.*, 2010 ▶). Hence, the binding interaction of the α-­amino/α-carboxy moiety of the substrate, the reaction intermediate and the inhibitor (acivicin) were common among all of the bacterial GGTs. In contrast, the environments surrounding the dihydroisoxazole ring of acivicin adducts differ among the GGTs.

Firstly, the interactions between the oxyanion hole (the main-chain amide NH) and the N2 atom in the dihydro­isoxazole ring were variable. In *B. subtilis* and *E. coli* GGT, the N2 atom and one of the oxyanion glycines (Gly485 in *B. subtilis* GGT and Gly483 in *E. coli*) formed a hydrogen bond *via* a water molecule (Figs. 3 ▶ *b* and 3*c*, respectively). In particular, this water was tightly fixed in this position in *E. coli* GGT since this water molecule was held in a short and hence tight hydrogen-bonding network, with the distances between the water to the N2 and the main-chain N of Gly483 being 2.6 and 2.7 Å, respectively (Fig. 3 ▶ *c*). In contrast, in *H. pylori* GGT no water molecule was observed in this position (Fig. 3 ▶ *d*). The distance between the N atom of Gly472 and the N2 atom is 3.4 Å, suggesting that Gly472 NH forms a weak hydrogen bond directly to the N2 atom or possibly makes no specific interaction. However, instead of the seemingly weak inter­action between Gly472 NH and the N2 atom in *H. pylori* GGT, another potential oxyanion glycine, Gly473, is located in such a position as to form a direct hydrogen bond to the N2 atom, with the distance between Gly473 N and N2 being 3.1 Å (Fig. 3 ▶ *d*). This hydrogen-bonding interaction is probably not available in the *B. subtilis* and *E. coli* GGTs because the corresponding atoms (N of Gly486 and N of Gly484, respectively) are not close enough to the N2 (≥3.4 Å) to assure a hydrogen-bonding interaction.

Secondly, the interactions between the lid-loop and the O1 atom of the dihydroisoxazole ring are different. In the *E. coli* and *H. pylori* GGTs, the lid-loop has been shown to cover the catalytic pocket and shield it from access of the external solvent when a substrate or inhibitor occupies the active site (Figs. 3 ▶ *c* and 3 ▶ *d*). The tyrosine residue on the tip of the lid-loop located near the O1 atom plays a key role as typically observed in *E. coli* GGT; the O^η^ atom of tyrosine (Tyr444) forms a hydrogen bond (with a distance of 3.2 Å) to the O1 atom in the dihydroisoxazole ring so that this interaction affects the orientation of the dihydroisoxazole ring (Fig. 3 ▶ *c*). In *H. pylori* GGT, the corresponding O atom of tyrosine (Tyr433) is located 4.7 Å from the O1 atom (Fig. 3 ▶ *d*). In the case of *B. subtilis* GGT, however, the lid-loop region was absent (Wada *et al.*, 2010 ▶) and thus the dihydroisoxazole ring is solvent-exposed (Fig. 3 ▶ *b*).

Finally, the C-terminal region of the S subunit appears to affect the binding of acivicin. In *H. pylori* GGT, the region intrudes into the catalytic pocket (Fig. 3 ▶ *d*); the side chain of Phe567 is located near the dihydroisoxazole ring, making a hydrophobic environment. This structural feature is consistent with direct interaction between the oxyanion-hole glycine NH and the N2 atom without an intervening water molecule, because in such a hydrophobic environment the water molecule as observed in the *B. subtilis* and *E. coli* GGTs would be unstabilized and its accommodation would seem to be highly unfavourable.

Consequently, in the bacterial GGTs the α-amino/α-­carboxy groups of the acivicin adduct are recognized and fixed by similar residues in almost the same manner by many hydrogen bonds and electrostatic interactions. In contrast, the environments surrounding the dihydroisoxazole ring of the acivicin adducts differ among the GGTs, resulting in diverse binding modes of the dihydroisoxazole ring and its C=N group that corresponds to the γ-carboxy group of glutamic acid. The significant diversity in the orientation of the dihydroisoxazole ring is thus caused by differences in the distance from the oxyanion hole (the NH of glycine), the interaction with the lid-loop (the O^η^ atom of a tyrosine residue) and the hydrophobic environment imposed by the C-terminal region of the S subunit (the side chain of Phe567). Despite each crystal belonging to a different space group and the crystal packing, the acivicin-binding mode is not likely to affect the crystal packing in each bacterial GGT because the catalytic pocket (the acivicin-binding site) was located far from the molecular surfaces of GGT protein that contribute to crystal formation.

### 3.4. Implications of the acivicin binding process in *B. subtilis* GGT by time-lapse soaking   

To gain insights into the binding affinity of the acivicin moiety, we assessed the electron-density maps after soaking the GGT crystals in acivicin solution for various soaking times and trapping its binding processes by flash-cooling.

**Figure 4:**
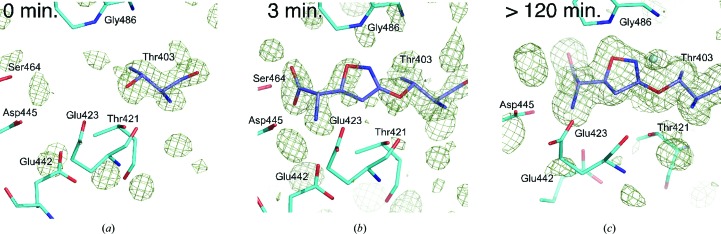
The electron density of the active pocket of *B. subtilis* GGT by time-lapse soaking. (*a*) OMIT map of the active pocket without soaking. The *F*_o_ − *F*_c_ map at 2.5σ is overlaid on the stick model, in which Thr403, the catalytic nucleophile residue, was omitted in the map calculation. (*b*) OMIT map of the crystal soaked in 5 m*M* acivicin solution for 3 min. The *F*_o_ − *F*_c_ map at 2.5σ is overlaid on the stick model, in which the acivicin adduct and Thr403 were omitted in the map calculation. (*c*) OMIT map of the crystal soaked in 5 m*M* acivicin solution for 120 min. The *F*_o_ − *F*_c_ map at 2.5σ is overlaid on the stick model, in which the acivicin adduct and Thr403 were omitted in the map calculation. The water molecule between Gly486 and acivicin adduct is indicated as a cyan ball. The view is rotated by 20° around the vertical axis relative to (*a*) and (*b*).

The OMIT *F*_o_ − *F*_c_ map obtained without soaking (acivicin-free form) gave weak/ambiguous electron density in the active pocket (Fig. 4 ▶ *a*), suggesting that the pocket is partially occupied by a small molecule such as a γ-glutamyl compound (GSH and/or glutamate) during the protein preparation. A similar vague electron density was also observed in the substrate-free *E. coli* GGT structure (Okada *et al.*, 2006 ▶). After 3 min of soaking in the acivicin solution (Fig. 4 ▶ *b*) the electron density clearly changed; electron density (>4σ in the OMIT map) located near Ser464, Asp445 and Glu442 appeared. This newly observed electron density is derived from the α-amino/α-carboxy moiety of acivicin. In contrast, the electron density corresponding to the five-membered dihydroisoxazole ring was almost invisible at this stage, indicating that the *B. subtilis* GGT first recognized the α-amino/α-­carboxy groups in preference to the distal dihydroisoxazole ring. However, the electron density of the dihydroisoxazole ring became apparent after soaking for more than 2 h (Fig. 4 ▶ *c*). In our previous study regarding the X-ray crystal structural analysis of *E. coli* GGT (Okada *et al.*, 2006 ▶), we trapped the enzyme–substrate intermediate (γ-glutamyl-enzyme complex) by the cryo-trapping method. In this experiment, the whole γ-­glutamyl group including the α-amino/α-carboxy moiety was clearly observed after a few minutes of soaking of the crystals in glutathione solution at ambient temperature. Thus, the partial electron-density maps obtained in the present study suggest that the binding process of acivicin to *B. subtilis* GGT is significantly slower compared with that of the substrate and is thought to be composed at least of two stages: in the early stage, the α-amino/α-carboxy groups of the acivicin molecule are rapidly bound (with high affinity) in the active pocket, and in the late stage the dihydroisoxazole ring is fixed by a slow reaction (with low affinity), perhaps concomitant with formation of the covalent bond between the acivicin C3 atom and Thr403 O^γ^.

As mentioned above, the GGTs specifically and strictly recognized the α-amino/α-carboxy moiety of the substrate/product by many hydrogen bonds, and the residues involved in the recognition of the α-amino/α-carboxy moiety are highly conserved among all GGTs. Since acivicin is an analogue of glutamate, *B. subtilis* GGT as well as all bacterial GGTs probably recognize the α-amino/α-carboxy in acivicin rapidly in preference to the other part of the molecule. However, the recognition and binding of the distal dihydroisoxazole ring are much less favourable owing to its significant structural differences between the substrate (a γ-glutamylamide) and the inhibitor (an imidoyl chloride). Considering that the binding of acivicin is accompanied by nucleophilic substitution at the C3 imidoyl C atom by the catalytic Thr residue, the affinity of acivicin is most significantly affected by the orientation of the electrophilic C=N group, which is constrained in a five-membered dihydroisoxazole ring; the orientation of C3 and N2 of acivicin (C=N) towards the catalytic residue (Thr O^γ^) and the oxy­anion hole (NH of Gly), respectively, was considerably different from the γ-carbonyl C and O (C=O) of the substrates in the catalyzed reaction of GGT. Therefore, the results of time-lapse soaking in this study reflect the loose recognition of the dihydroisoxazole moiety of acivicin, and the structural constraint by the five-membered ring is one of the factors that causes the diversity in the orientations of the bound dihydroisoxazole ring among GGTs.

## 4. Discussion   

Acivicin is a well known glutamine antagonist that irreversibly inhibits a wide range of Gln amidotransferases (GATs), which are glutamine-utilizing biosynthetic enzymes for purine, pyrimidine, hexosamines and amino acids (O’Dwyer *et al.*, 1984 ▶; Earhart & Neil, 1985 ▶). Since the discovery of inhibition of GGT by acivicin (Reed *et al.*, 1980 ▶), this compound has been widely used for the inhibition of GGT in *in vitro* and *in vivo* experiments over the past three decades; to the best of our knowledge, more than 190 scientific papers have been reported to date on the inhibition of GGT by acivicin. However, X-ray structural study of the reaction mechanism and the binding mode has been completely lacking, despite the structural uniqueness of acivicin and its notorious *in vivo* biological activity. Previous studies on mammalian GGTs by isotopically labelled acivicin gave seemingly odd results: the residue to which the inhibitor was bound was Thr523 for rat kidney GGT (Stole *et al.*, 1990 ▶, 1994 ▶), whereas the residue was Ser405 for porcine kidney GGT (Smith *et al.*, 1995 ▶) and Ser406 for human kidney GGT (Smith *et al.*, 1995 ▶), none of which are the catalytic Thr residue of mammalian enzymes, which is essential for catalysis and conserved among GGTs. More recently, it was reported that acivicin was not bound in the active site of recombinant human GGT, at least in the same manner as the donor substrate (a γ-glutamyl compound; Castonguay *et al.*, 2007 ▶). These results suggested that acivicin may, at least for mammalian GGTs, not act as an active-site-directed inhibitor and that the inhibition of GGT by acivicin was a fortuitous event that was brought about by the presence of an electrophilic functionality (an imidoyl chloride) near the γ-carboxy of glutamate. If this is the case, the mode of action of acivicin should not be uniform toward GGTs and it may bind to the enzyme in different manners.

In this study, we compared the binding mode of the acivicin adduct among three GGTs and illustrated the diversity in the manner of binding, especially in the dihydroisoxazole ring (Fig. 3 ▶). The results revealed that, at least in bacterial GGTs, the α-amino/α-carboxy moiety of acivicin served as a major recognition element (Fig. 4 ▶), but not the dihydroisoxazole ring. The results of this study also tell us that the active-pocket structures of bacterial GGTs are substantially different from each other, not to mention from the mammalian enzymes, and at least are not ideal to accommodate the structurally constrained dihydroisoxazole ring of acivicin. This might be a reason why acivicin is a rather weak inhibitor of human GGT, with the rate of inactivation of human GGT being more than 10^4^ times slower than that of the *E. coli* enzyme (Han *et al.*, 2007 ▶). Further studies of the individual GGTs, including the human enzyme, are crucial for understanding the structures and reaction mechanisms and thus the physiological roles of GGT. This is our first step towards an understanding of the mode of action of acivicin towards hitherto structurally unidentified mammalian GGTs. An X-ray crystal structure of glutamate-bound human GGT was published during submission of this article. The details of the Cys-Gly binding site are not yet defined (West *et al.*, 2013 ▶). The chemistry and properties of acivicin studied by the X-ray crystallographic analysis of GGT are also expected to shed light on the reaction and chemistry of acivicin with glutamine amidotransferases, the natural target of acivicin, in which the catalytic Cys residue is modified.

## Supplementary Material

PDB reference: γ-glutamyltranspeptidase, 3whq

PDB reference: soaked with acivicin, 3whr

PDB reference: 3whs

## Figures and Tables

**Table 1:** Crystallographic data, data-collection and refinement statistics Values in parentheses are for the outermost shell.

Soaking time	0 min	3 min	120 min
PDB code	3whq	3whr	3whs
Crystallographic data			
Space group	*P*2_1_2_1_2_1_	*P*2_1_2_1_2_1_	*P*2_1_2_1_2_1_
Unit-cell parameters (Å)	*a* = 59.6, *b* = 71.7, *c* = 143.7	*a* = 58,8, *b* = 71.8, *c* = 142.4	*a* = 60.1, *b* = 71.7, *c* = 144.4
Resolution range (Å)	50–1.85 (1.92–1.85)	50–1.58 (1.64–1.58)	50–1.80 (1.86–1.80)
Unique reflections	56570	81531	56723
Mean *I*/σ(*I*)	12.3	21.7	13.1
Multiplicity	8.1 (7.8)	7.4 (7.1)	7.3 (7.4)
Completeness (%)	99.9 (100)	98.1 (97.8)	96.8 (92.6)
*R*_merge_ † (%)	6.2 (33.9)	4.0 (17.0)	5.8 (39.6)
Refinement statistics			
*R*_cryst_ ‡ (%)	17.6	16.8	18.2
*R*_free_ § (%)	21.4	18.4	21.0
Disordered regions¶			
L subunit	36, 397–402	36, 396–402	36, 396–402
S subunit	587	586–587	586–587
R.m.s. deviations from ideal values			
Bond length (Å)	0.014	0.008	0.013
Bond angle (°)	1.4	1.2	1.4
Average *B* factor (Å^2^)	20.0	15.3	24.0
Ramachandran plot			
Most favoured (%)	91.0	91.2	91.4
Additionally allowed (%)	8.7	8.5	8.3
Generously allowed (%)	0.0	0.0	0.0
Disallowed†† (%)	0.2	0.2	0.2

† *R*_merge_ = 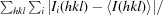 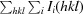, where 〈*I*(*hkl*)〉 is the average intensity over equivalent reflections.

‡ *R*_cryst_ = 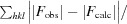 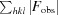.

§ *R*_free_ is the *R* value calculated for 5% of the data set which was not included in the refinement.

¶ The numbers shown are those of invisible residue.

†† Glu423, which corresponds to Asn411 in *E. coli* GGT, was in the disallowed region in all GGT structures.
